# Serum NfL and GFAP in post-COVID syndrome: minimal evidence of CNS injury after adjusting for confounders

**DOI:** 10.3389/fncel.2026.1750121

**Published:** 2026-03-20

**Authors:** Michael Wunderle, Andrea Ribeiro, Isabelle Lethen, Rebecca Wicklein, Emily Feneberg, Anna Wöhnl, Johanna Negele, Veronika Kesseler, Samuel Niedermayer, Maciej Lech, Timon Wallraven, Christoph Schmaderer

**Affiliations:** 1 Department of Nephrology, TUM School of Medicine and Health, TUM University Hospital, Technical University of Munich, Munich, Germany; 2 Department of Neurology, TUM School of Medicine and Health, TUM University Hospital, Technical University of Munich, Munich, Germany; 3 Medizinische Klinik Und Poliklinik IV, LMU University Hospital Munich, Munich, Germany; 4 German Centre for Infection Research (DZIF), Munich Partner Site, Munich, Germany

**Keywords:** CNS injury, glial fibrillary acidic protein (GFAP), Long COVID, neurofilament light chain (NFL), post-COVID syndrome

## Abstract

**Background:**

Post-COVID syndrome (PCS) often includes neurological symptoms, but evidence for persistent CNS injury remains inconsistent. Serum neurofilament light chain (NfL) and glial fibrillary acidic protein (GFAP) are biomarkers of neuronal and astroglial injury. We investigated whether serum NfL and GFAP differ between PCS patients and recovered controls after adjusting for age and renal function.

**Methods:**

In this prospective single-center case–control study, serum NfL and GFAP were quantified using Simoa® (Quanterix) in 102 PCS patients and 102 recovered controls. Group comparisons employed Mann–Whitney tests and ANCOVA-style multivariable linear regression of log-transformed biomarkers adjusted for age, sex, and eGFR. Associations with eGFR were examined in multivariable models, and findings were validated in an age- and sex-matched cohort.

**Results:**

Age emerged as the primary determinant of NfL and GFAP concentrations. The inverse correlations with renal function (NfL *ρ* = −0.23; GFAP *ρ* = −0.33) and the initially higher GFAP in PCS (60.4 vs. 52.3 pg/mL; *p* = 0.002) were largely explained by age. After adjustment for age, sex, and eGFR, neither biomarker showed independent differences between groups (adjusted GMRs: NfL 1.04 [0.91–1.18], *p* = 0.59; GFAP 1.10 [0.96–1.26], *p* = 0.15). In an age- and sex-matched cohort (71 pairs), adjusted analyses confirmed no difference in NfL (*p* = 0.48), while GFAP demonstrated a significant increase in PCS (*β* = 0.15, *p* = 0.025).

**Conclusion:**

GFAP concentrations were modestly elevated in PCS in an age- and sex-matched cohort and persisted after adjustment for kidney function, whereas NfL showed no group differences. These findings argue against widespread neuroaxonal injury in PCS and suggest only a subtle astroglial signal in a subset of patients. Rigorous adjustment for confounders—particularly age, sex, and renal function—is essential for valid interpretation of serum neuroinjury biomarkers in PCS.

## 1 Introduction

Post-COVID syndrome (PCS), also termed post-acute sequelae of SARS-CoV-2 (PASC) or Long COVID, encompasses a constellation of persistent or relapsing symptoms beyond the acute phase and spans unexplained symptom clusters as well as incident medical diagnoses across organ systems (Nalbandian et al., 2021; Taquet et al., 2021; National Academies of Sciences, 2024). Clear but variably applied definitions from WHO, CDC, NIH, and national bodies complicate case ascertainment and contribute to heterogeneity across studies (Peluso and Deeks, 2024). Neurological complaints—fatigue, cognitive dysfunction, sleep disturbance, and mood symptoms—feature prominently in PCS and are supported by multimodal imaging and physiological evidence suggesting subtle central nervous system (CNS) involvement (Sollini et al., 2021; Hellgren et al., 2021; Antony and Haneef, 2020; Talkington et al., 2024). Mechanistic hypotheses include neuroinflammation, endothelial dysfunction with blood–brain barrier disruption, microvascular injury, and dysautonomia, any of which could theoretically produce measurable signals in fluid biomarkers of CNS injury (Peluso and Deeks, 2024; Talkington et al., 2024; Möller et al., 2023; Greene et al., 2024; Bohmwald et al., 2018; Kempuraj et al., 2024b; Kempuraj et al., 2024a; Silva Andrade et al., 2021; Wang et al., 2022).

Neurofilament light chain (NfL) and glial fibrillary acidic protein (GFAP) are established blood biomarkers of axonal and astroglial injury that became accessible in serum through ultrasensitive single-molecule assays (Andreasson et al., 2016; Peluso et al., 2022; Bark et al., 2023; Plantone et al., 2024; Park et al., 2024). NfL, a structural component of the axonal cytoskeleton, is released following axonal injury or degeneration (Fusco et al., 2025), whereas GFAP, an astrocytic intermediate filament protein, is upregulated and released during astroglial activation or reactive astrogliosis (Yang and Wang, 2015). Both biomarkers are therefore considered indicators of neuroaxonal and astroglial pathology in conditions associated with neuroinflammatory, vascular, or blood–brain barrier-related mechanisms. In acute COVID-19, elevated NfL and GFAP have been repeatedly reported, often correlating with disease severity and mortality (Aamodt et al., 2021; Sievert et al., 2023; Sahin et al., 2022). Whether such injury signals persist into PCS is unsettled. While some studies and systematic reviews reported altered NfL or GFAP levels in subsets of PCS patients, particularly those with neurological symptoms (Lai et al., 2023; Boesl et al., 2024), others found concentrations within age-expected ranges and no association with cognitive performance (Boesl et al., 2024). Taken together, existing studies provide conflicting evidence regarding persistent neuroaxonal or astroglial injury in PCS. This heterogeneity raises the possibility that reported differences in NfL and GFAP may partly reflect variability in study design, case definitions, and insufficient adjustment for key biological confounders rather than ongoing CNS injury

Interpretation of serum NfL and GFAP levels critically depends on biological confounders. NfL increases with age even in healthy individuals and is associated with renal function, while GFAP has also been reported to rise with ageing and impaired kidney function (Akamine et al., 2020; Polymeris et al., 2022; Hviid et al., 2020; Benkert et al., 2022; Yilmaz et al., 2017)

Inflammatory state and hypoxemia, particularly during acute illness, may further influence circulating concentrations (Abdelhak et al., 2025; De Lorenzo et al., 2024; Huang et al., 2023). Inadequate adjustment for these factors may therefore obscure or falsely suggest disease-related biomarker differences in PCS.

In PCS, persistent symptoms often lack a unifying structural lesion, and prevailing mechanistic frameworks emphasize immune dysregulation, endothelial dysfunction, and autonomic disturbances rather than ongoing cytodestructive injury (Boesl et al., 2024; Kanberg et al., 2024; Greenhalgh et al., 2024). This may explain why many symptomatic patients exhibit normal or near-normal NfL and GFAP concentrations, while reported elevations in some cohorts may reflect residual convalescent biology, comorbidities, or unmeasured confounding rather than active neuronal or astrocytic degeneration. The resultant literature is therefore mixed: studies enriched for early convalescent sampling or lacking comprehensive adjustment often report higher NfL or GFAP levels, whereas well-phenotyped PCS cohorts more frequently show values within age-expected ranges. Against this backdrop, robust evaluation of NfL and GFAP as markers of persistent neurobiological injury in PCS requires careful case definition and explicit adjustment for key confounders, particularly age and renal function.

Notably, prior PCS biomarker studies rarely combined comprehensive multivariable confounder adjustment with matched sensitivity analyses, limiting the interpretability of reported group differences. Accordingly, a rigorously controlled re-evaluation of NfL and GFAP in well-defined PCS cohorts is required to disentangle disease-related signals from methodological and biological confounding.

We aimed to determine whether serum NfL and GFAP differ between PCS patients and COVID-19–recovered controls after rigorous adjustment for age, sex, and renal function, and to test the robustness of these comparisons using age- and sex-matched sensitivity analyses. By additionally quantifying the independent contributions of age, sex, and estimated glomerular filtration rate (eGFR) to biomarker variability, this study provides a methodologically distinct re-assessment of NfL and GFAP in PCS patients.

## 2 Methods

### 2.1 Study design and cohort

This work is part of the “All Eyes on PCS” study, a prospective, observational single-center investigation aimed at assessing retinal microvascular changes in PCS and providing a detailed clinical characterization of affected patients. A comprehensive description of recruitment procedures and clinical assessments has been published previously (Kuchler et al., 2023).

Participants were eligible if they had a confirmed SARS-CoV-2 infection (PCR or antibody evidence ≥3 months prior) and experienced persistent PCS-related symptoms for at least 2 months without an alternative medical explanation. Exclusion criteria included absence of informed consent, age below 18 years, pregnancy, active malignancy, conditions with markedly reduced life expectancy, rheumatologic autoimmune diseases, as well as ocular or neurological comorbidities such as cataract, glaucoma, or epilepsy.

Between October 2022 and September 2023, 105 individuals were recruited – 76 via social media and 29 from the PCS outpatient clinic. Three participants were excluded from the final analysis: one due to missing infection verification, one for lacking a temporal relationship between infection and symptom onset, and one because PCS-typical symptoms had resolved by the time of inclusion. PCS symptom severity was heterogeneous across participants and is illustrated descriptively using PCS Score and C19-YRS severity distributions (Supplementary Figure 6). Controls who had fully recovered from a previous COVID-19 infection were recruited between December 2022 and January 2025.

All examinations were performed at the Technical University of Munich (Departments of Nephrology, Neurology, and Ophthalmology) by experienced investigators who were aware of the study framework but not involved in hypothesis generation or statistical evaluation. The study protocol was approved by the local ethics committee (Ethics Committee of the Technical University of Munich, School of Medicine, Klinikum rechts der Isar; approval number: 2022-317-S-SR) and prospectively registered (ClinicalTrials.gov identifier: NCT05635552). All procedures adhered to the Declaration of Helsinki and institutional ethical standards. Written informed consent was obtained from all participants prior to study inclusion.

### 2.2 NfL and GFAP measurement

Serum concentrations of NfL and GFAP were quantified using the Single Molecule Array (Simoa^®^) technology on the HD-X Analyzer platform (Quanterix Corp., Billerica, MA, USA). Blood samples were collected by trained research staff during morning hours (before noon) using standard serum collection tubes. Following collection, samples were centrifuged immediately according to local standard operating procedures, and serum aliquots were stored at −80 °C until analysis.

All samples were measured after a single freeze–thaw cycle. No additional freeze–thaw cycles occurred prior to or during analysis. Measurements were performed with the commercially available Simoa^®^ Neurology 2-Plex B Advantage Kit (Quanterix), strictly following the manufacturer’s instructions. Calibration was conducted using a four-parameter logistic (4PL) curve fit with 1/y^2^ weighting. The calibration range was approximately 0–500 pg./mL for NfL and 0–10 ng/mL for GFAP. Calibrators were run in triplicate with a total volume of 330 μL per analyte on a 96-well plate. Lower Limits of Quantification were set according to the manufacturer’s specifications (GFAP: 16.6 pg./mL NfL: 0.800 pg./mL).

Inter- and intra-assay variability were not formally assessed in this study. Repeat analyses were limited to quality-control purposes in a small number of samples (three samples initially below the lower limit of quantification and one sample excluded due to visible precipitation in the assay well). Laboratory staff were blinded to clinical and imaging data.

## 3 Statistical analysis

### 3.1 Data preparation and quality control

All variables were systematically checked prior to statistical analyses (Supplementary Figures 1–3). Serum NfL and GFAP displayed right-skewed distributions and were therefore log-transformed for regression modeling. Potential outliers were identified using Tukey’s fences and visualized with boxplots. Outliers were not removed from the primary analyses given their likely biological origin, but robustness was evaluated in sensitivity analyses. Missing data were quantified and patterns visualized using naniar. NfL and GFAP showed approximately 10% missingness, and eGFR ~6%. Little’s MCAR test suggested that data were not missing completely at random (*p* = 0.016). Consequently, all primary analyses were conducted as complete-case analyses, with sensitivity analyses using alternative approaches to assess robustness (Supplementary Tables 1–4).

### 3.2 Univariate group comparisons

For the primary descriptive comparison of PCS versus HC, crude differences in serum NfL and GFAP were assessed using Mann–Whitney U tests. Effect sizes were reported as Hodges-Lehmann median differences with 95% confidence intervals. In sensitivity analyses, log-transformed biomarkers were compared using Welch’s t-tests, with results reported as geometric mean ratios (GMRs) and 95% CIs. *p*-values from univariate comparisons were adjusted for multiple testing using the Holm method (Supplementary Table 5).

### 3.3 Multivariable models

The primary inferential analyses were ANCOVA-style linear regression models with log-transformed biomarkers as dependent variables. Group status (PCS vs. HC) was the main predictor, adjusted for the pre-specified confounders age, sex, and eGFR, which were selected *a priori* based on their well-established, direct effects on circulating NfL and GFAP concentrations. Additional clinical variables such as comorbidities, body mass index, vaccination status, or acute COVID-19 severity were not included to avoid overadjustment, as their role as independent confounders of circulating biomarker concentrations in PCS is less well established. Given the moderate sample size, the number of covariates in multivariable models was intentionally restricted to preserve model stability and to reduce the risk of overfitting. Regression coefficients were reported as *β* estimates on the log scale and exponentiated to geometric mean ratios (PCS/HC) with 95% CIs. Model fit was summarized with R^2^ and adjusted R^2^. Variance inflation factors (VIFs) were computed to assess multicollinearity; VIF > 5 was considered problematic.

### 3.4 Diagnostics

Residual distributions were inspected with Q-Q plots, residual-fitted value plots, and scale-location plots (Supplementary Figures 4, 5). Cook’s distances and leverage values were assessed to identify influential observations. Robust standard errors (HC3) were calculated as sensitivity checks for heteroscedasticity, results were unchanged.

### 3.5 Sensitivity analyses

Robustness of the main conclusions was examined in several pre-specified sensitivity analyses:

- Alternative renal metric: replacing eGFR with log-transformed serum creatinine (Supplementary Table 1)
- Non-linearity: modelling eGFR using restricted cubic splines (3–4 knots) (Supplementary Table 2)
- CKD exclusion: repeating models after excluding participants with eGFR <60 mL/min/1.73 m^2^ (Supplementary Table 3)
- Age stratification: exploratory subgroup analyses stratified by median age and tertiles (Supplementary Table 4)
- Multiple comparisons: descriptive univariate *p*-values adjusted using Holm’s method (Supplementary Table 5)

### 3.6 Transparency and reproducibility

The primary endpoint was the adjusted group effect (PCS vs. HC) for each biomarker on the log scale. Confounders were pre-specified (age, sex, eGFR) based on clinical considerations, given the known impact of renal function on neurofilament levels. Transformations (log) and statistical methods (non-parametric tests for descriptive robustness) were specified a priori. Exclusion criteria included implausible biomarker values, missingness in key covariates, and hemolysis. All analyses were conducted in R (version 2024.12.1 + 563) and all analysis scripts were archived for reproducibility.

## 4 Results

### 4.1 Baseline demographics of the cohort

The study included 102 PCS patients and 102 recovered controls (Table 1). PCS participants were older (median 41.5 vs. 30.0 years, *p* < 0.001) with a similar sex distribution (female 75.5% vs. 71.6%, *p* = 0.63). BMI and obesity were comparable (BMI 23.7 vs. 22.8 kg/m^2^, *p* = 0.15; obesity 15.7% vs. 7.8%, *p* = 0.13), as were nicotine abuse rates (*p* = 0.52). Hypertension (*p* = 0.019) and hypercholesterolemia (*p* < 0.001) were more frequent in PCS. Acute infection severity and viral variant distributions differed between groups (*p* < 0.001 and *p* = 0.002, respectively), while the number of infections was similar (*p* = 0.11). Time since infection was shorter in PCS (462.2 ± 267.6 vs. 555.0 ± 358.9 days, *p* = 0.041). Vaccination counts and vaccination before infection differed markedly (both *p* < 0.001). Myalgic Encephalomyelitis/Chronic Fatigue Syndrome (ME/CFS) was present in 61.4% of PCS and absent in controls (*p* < 0.001). Kidney function was slightly lower in PCS (eGFR 96.9 vs. 103.9 mL/min/1.73 m^2^, *p* = 0.002), while creatinine was similar (*p* = 0.77). For biomarkers, NfL did not differ (4.9 vs. 4.8 pg./mL, *p* = 0.14), whereas GFAP was higher in PCS (60.4 vs. 52.3 pg./mL, *p* = 0.002).

**Table 1:** Baseline characteristics of the full cohort (PCS vs. COVID-19 recovered controls).

Variable	COVID-19 recovered (*n* = 102)	PCS patients (*n* = 102)	*p*-value
Age (years), median (IQR)	30.0 (25.0–37.8)	41.5 (32.0–52.0)	<0.001
Sex, female *n* (%)	73 (71.6)	77 (75.5)	0.63
BMI (kg/m^2^), median (IQR)	22.8 (21.1–24.7)	23.7 (21.0–27.0)	0.15
Obesity, *n* (%)	8 (7.8)	16 (15.7)	0.13
Nicotine abuse, *n* (%)	14 (13.7)	10 (9.8)	0.52
Hypertension, *n* (%)	5 (4.9)	16 (15.7)	0.019
Diabetes mellitus type II, *n* (%)	0 (0.0)	1 (1.0)	1.0
Hypercholesterolemia, *n* (%)	21 (22.1)	46 (46.5)	<0.001
Severity of acute infection (ordinal), *n* (%)			<0.001
0	1 (1.0)	0 (0.0)	
1	9 (8.8)	1 (1.0)	
2	81 (79.4)	61 (59.8)	
3	10 (9.8)	34 (33.3)	
4–8	1 (1.0)	7 (6.9)	
SARS-CoV-2 variant, *n* (%)			0.002
Unknown	80 (79.2)	63 (61.8)	
Alpha	1 (1.0)	7 (6.9)	
Delta	2 (2.0)	13 (12.7)	
Omicron	18 (17.8)	19 (18.6)	
Number of infections, *n* (%)			0.11
1	62 (60.8)	71 (69.6)	
2	31 (30.4)	29 (28.4)	
≥3	10 (9.8)	3 (2.0)	
Time since infection (days), mean ± SD	555.0 ± 358.9	462.2 ± 267.6	0.041
Number of vaccinations, *n* (%)			<0.001
0–1	1 (1.0)	9 (8.8)	
2	8 (7.9)	27 (26.5)	
≥3	93 (91.1)	66 (64.7)	
Vaccinated before infection, *n* (%)			<0.001
Yes	94 (94.0)	71 (72.4)	
ME/CFS, *n* (%)	0 (0.0)	62 (61.4)	<0.001
eGFR (ml/min/1.73 m^2^), median (IQR)	103.9 (92.2–119.0)	96.9 (84.7–108.4)	0.002
Creatinine (mg/dl), mean ± SD	0.8 ± 0.1	0.8 ± 0.1	0.77
NfL (pg/ml), median (IQR)	4.8 (3.6–6.0)	4.9 (4.0–8.0)	0.14
GFAP (pg/ml), median (IQR)	52.3 (36.7–64.4)	60.4 (45.0–81.9)	0.002

*N* = 102 per group. Continuous variables are shown as median (IQR) or mean ± SD (as indicated); categorical variables as n (%). *p*-values were derived from ANOVA (parametric), Kruskal-Wallis (non-parametric), and χ^2^ or Fisher’s exact tests for categorical/proportional data, as appropriate.

### 4.2 NfL and GFAP distributions between cohorts

**Figure 1:**
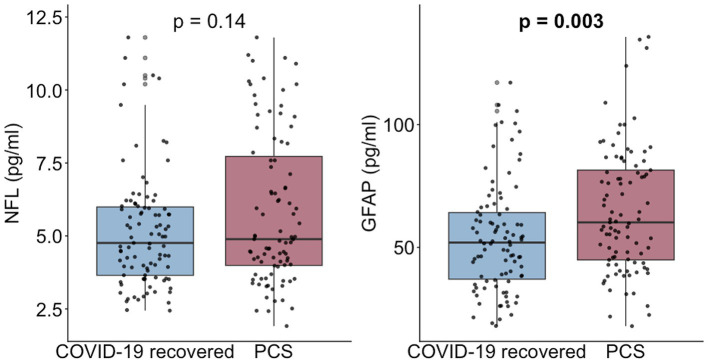
NfL and GFAP concentrations in PCS and recovered controls. Boxplots display medians and interquartile ranges with individual data points overlaid. *p*-values derived from Wilcoxon test.

In unadjusted analyses, serum concentrations of NfL did not differ significantly between individuals with PCS and recovered controls (median [IQR] 4.89 [3.99–8.02] pg./ml vs. 4.76 [3.65–6.02] pg./ml; *p* = 0.14; Figure 1). By contrast, GFAP concentrations were significantly higher in the PCS group than in controls (60.4 [45.0–81.9] pg./ml vs. 52.3 [36.7–64.4] pg./ml; *p* = 0.002).

**Figure 2:**
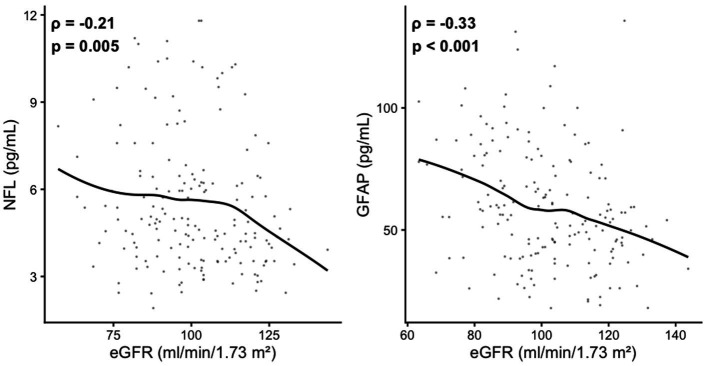
Scatterplots of serum NfL and GFAP in relation to kidney function (eGFR) across the entire cohort. Each dot represents an individual participant; solid lines indicate locally weighted regression (LOESS). Both NfL and GFAP were inversely correlated with eGFR.

Across the entire cohort, both serum NfL and GFAP concentrations correlated inversely with kidney function. NfL was negatively associated with eGFR (*ρ* = −0.23; *p* = 0.003; Figure 2), and GFAP showed an even stronger negative association (ρ = −0.33; *p* < 0.001). These findings indicate that higher biomarker concentrations are linked to lower renal function, highlighting the importance of adjusting for eGFR in subsequent analyses.

### 4.3 Primary univariate group comparisons

In unadjusted comparisons (Table 2), NfL concentrations did not differ significantly between PCS and recovered controls (Mann–Whitney *p* = 0.14; Hodges-Lehmann median difference 0.11 [95% CI 0.01–0.24]; GMR 0.90 [95% CI 0.80–1.01]; *p* = 0.075). By contrast, GFAP concentrations were significantly higher in PCS compared with controls (Mann–Whitney *p* = 0.0025; Hodges-Lehmann median difference 0.22 [95% CI 0.09–0.36]; GMR 0.82 [95% CI 0.73–0.93]; *p* = 0.0018).

**Table 2:** Univariate group comparisons of serum NfL and GFAP between patients with PCS and recovered controls.

Univariate group comparisons (PCS vs. HC)						
Marker	Mann–Whitney *p*	HL diff	HL 95% CI	GMR (PCS/HC)	GMR 95% CI	*t*-test *p*
NfL	0.140	0.109	[0.01; 0.24]	0.90	[0.8; 1.01]	0.075
GFAP	**0.002**	0.222	[0.09; 0.36]	0.82	[0.73; 0.93]	**0.002**

Results are presented as Mann–Whitney *p-*values, Hodges-Lehmann median differences with 95% confidence intervals (CI), and geometric mean ratios (GMR) with 95% CI from log-transformed analyses. Bold values indicate statistically significant *p*-values.

### 4.4 Association with kidney function

In multivariable models including age and sex (Table 3), the inverse associations of both NfL and GFAP with eGFR observed in unadjusted analyses were attenuated and no longer statistically significant (NfL: −2.5% [95% CI −6.8 to 2.1] per +10 mL/min eGFR, *p* = 0.29; GFAP: −3.6% [−8.0 to 1.1], *p* = 0.13). By contrast, GFAP concentrations remained independently associated with age (0.9% increase per year, 95% CI 0.2–1.5; *p* = 0.009), whereas NfL showed a non-significant trend towards higher values with increasing age (*p* = 0.074). These findings suggest that the apparent crude relationship between kidney function and biomarker concentrations is largely explained by age.

**Table 3:** Multivariable linear regression models of log-transformed NfL and GFAP.

Linear models on log scale: log (Biomarker)~eGFR + age + sex					
Marker	Term	Coefficient (log scale)			Percent change per +10 ml/min eGFR
		Estimate	95% CI	*p*-value	
GFAP	eGFR (per 1 mL/min)	−0.004	[−0.01; 0.00]	*p* = 0.128	−3.6% (−8%; 1.1%)
GFAP	Age (years)	0.009	[0.00; 0.02]	*p* = 0.009	
NfL	eGFR (per 1 ml/min)	−0.002	[−0.01; 0.00]	*p* = 0.287	−2.5% (−6.8%; 2.1%)
NfL	Age (years)	0.006	[−0.00; 0.01]	*p* = 0.074	

Models included eGFR, age, and sex as predictors.

### 4.5 Adjusted group comparisons

In multivariable ANCOVA-style linear regression models adjusting for age, sex, and eGFR, neither serum NfL nor GFAP showed independent differences between PCS and HC (Tables 4, 5). For NfL, the group coefficient (PCS vs. HC) was small and non-significant (*β* = 0.04, 95% CI −0.09 to 0.17, *p* = 0.59). NfL levels were also not independently associated with age (*β* = 0.01, 95% CI −0.002 to 0.012, *p* = 0.14) or eGFR (β = −0.003, 95% CI −0.007 to 0.002, *p* = 0.28). The model explained little variance (adj. R^2^ = 0.046).

**Table 4:** ANCOVA-style linear regression model for log-transformed NfL, adjusted for group (PCS vs. HC), age, sex, and eGFR.

Predictors	log(NfL)				
	Estimates	std. error	95% CI	Statistic	*p*
(Intercept)	1.68	0.32	[1.04; 2.32]	5.19	**<0.001**
Group: PCS (vs HC)	0.04	0.07	[−0.09; 0.17]	0.54	0.588
Age (years)	0.01	0.00	[−0.00; 0.01]	1.50	0.137
Male (vs female)	−0.02	0.07	[−0.16; 0.11]	−0.34	0.737
eGFR (ml/min/1.73 m^2^)	−0.00	0.00	[−0.01; 0.00]	−1.09	0.277
Observations	172				
R^2^/R^2^ adjusted	0.068/0.046				

Coefficients are shown on the log scale (β) with 95% confidence intervals.

**Table 5:** ANCOVA-style linear regression model for log-transformed GFAP, adjusted for group (PCS vs. HC), age, sex, and eGFR.

Predictors	log(GFAP)				
	Estimates	std. error	95% CI	Statistic	*p*
(Intercept)	4.12	0.33	[3.47; 4.77]	12.49	**<0.001**
Group: PCS (vs HC)	0.10	0.07	[−0.04; 0.23]	1.44	0.151
Age (years)	0.01	0.00	[0.00; 0.01]	1.97	0.051
Male (vs female)	−0.13	0.07	[−0.27; 0.00]	−1.97	0.050
eGFR (ml/min/1.73 m^2^)	−0.00	0.00	[−0.01; 0.00]	−1.56	0.119
Observations	174				
R^2^/ R^2^ adjusted	0.158/0.138				

Coefficients are shown on the log scale (β) with 95% confidence intervals.

For GFAP, no significant group effect was observed (*β* = 0.10, 95% CI −0.04 to 0.23, *p* = 0.15). GFAP showed a borderline positive association with age (*β* = 0.01, 95% CI 0.00 to 0.014, *p* = 0.051) and a negative association with male sex (β = −0.13, 95% CI -0.27 to 0.00, *p* = 0.050). The association with eGFR was inverse but not statistically significant (*β* = −0.004, 95% CI −0.008 to 0.001, *p* = 0.12). The adjusted R^2^ was 0.138.

**Figure 3:**
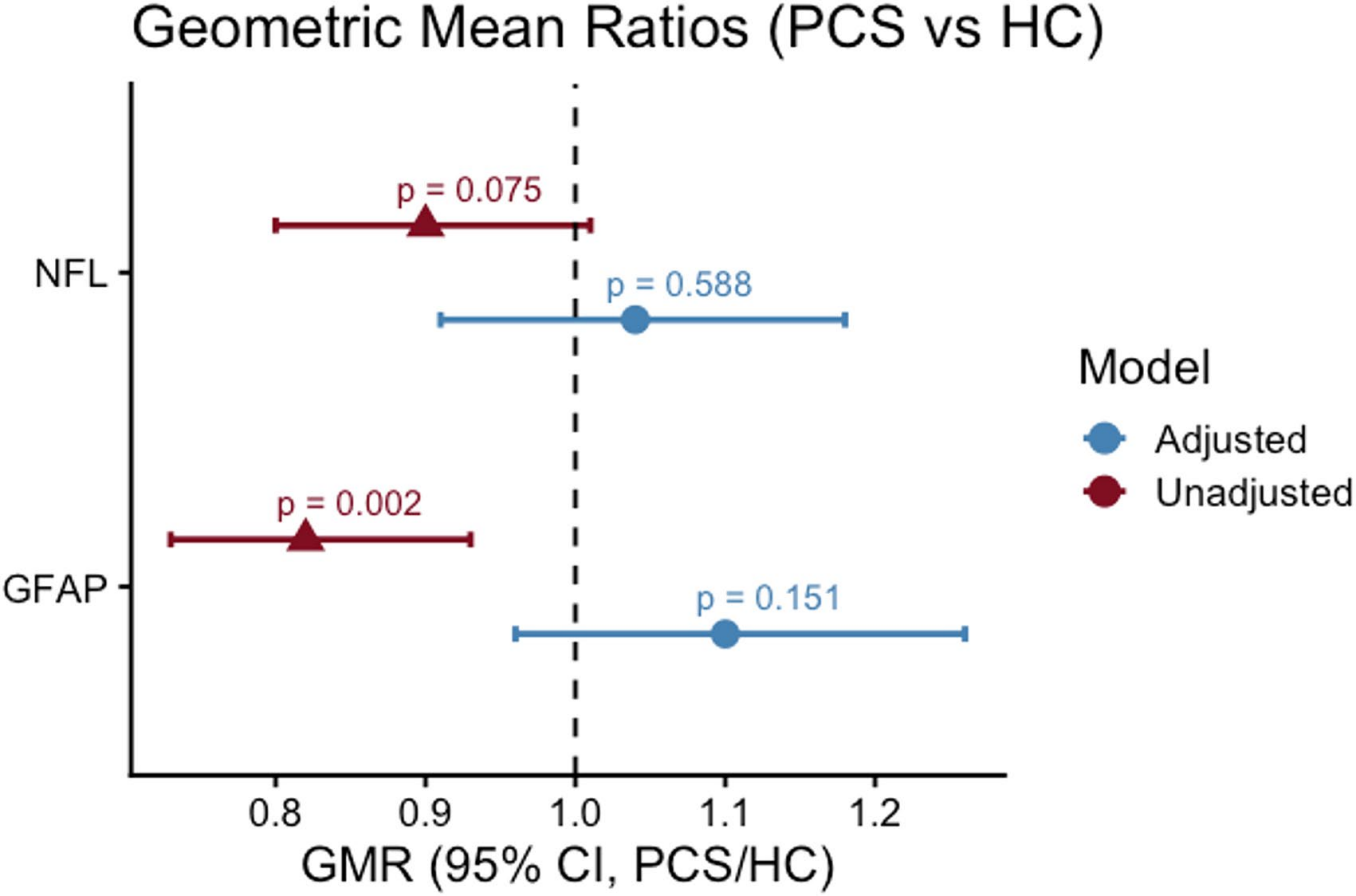
Forest plot of unadjusted and adjusted geometric mean ratios (PCS vs. COVID-19 recovered controls) for serum NfL and GFAP. Points represent geometric mean ratios (PCS/HC) with 95% confidence intervals, derived from log-transformed biomarker levels. Red symbols indicate unadjusted analyses, and blue symbols indicate models adjusted for age, sex, and eGFR. The dashed vertical line represents the null value (GMR = 1).

Crude and adjusted group effects are shown in Figure 3. In unadjusted analyses, serum GFAP concentrations were significantly higher in PCS patients compared with COVID-19 recovered controls (GMR 0.82 [95% CI 0.73–0.93]; *p* = 0.002), whereas NfL showed no significant group differences (GMR 0.90 [0.80–1.01]; *p* = 0.075). After adjustment for age, sex, and eGFR, neither biomarker differed significantly between groups (NfL: adjusted GMR 1.04 [0.91–1.18]; *p* = 0.588; GFAP: adjusted GMR 1.10 [0.96–1.26]; *p* = 0.151).

Variance inflation factors (all VIF < 2) indicated no evidence of problematic collinearity between age, sex, and eGFR. Model diagnostics did not reveal major violations of assumptions, and sensitivity analyses using robust standard errors confirmed the stability of findings (see Supplementary Figures 2–5). In adjusted linear models of the full cohort, ME/CFS status was not associated with either biomarker (Supplementary Table 8).

### 4.6 Age- and sex-matched analysis

After 1:1 age- and sex-matching, 71 PCS patients and 71 recovered controls were included (Table 6). Age was balanced (median 36.0 vs. 34.0 years, *p* = 0.37) and sex distributions were comparable (female 77.5% vs. 70.4%, *p* = 0.44). BMI was similar (23.6 vs. 23.0 kg/m^2^, *p* = 0.99). Kidney function did not differ (eGFR 98.9 vs. 98.2 mL/min/1.73 m^2^, *p* = 0.52; creatinine 0.8 ± 0.1 mg/dL in both groups, *p* = 0.87). Baseline NfL did not differ between groups (5.0 vs. 5.3 pg/mL, *p* = 0.48), whereas GFAP was higher in PCS (61.4 vs. 55.1 pg/mL, *p* = 0.016).

**Table 6:** Baseline characteristics of PCS patients and COVID-19 recovered controls after age- and sex-matching.

Variable	COVID-19 recovered (*n* = 71)	PCS patients (*n* = 71)	*p*-value
Age (years), median (IQR)	34.0 (28.0–45.5)	36.0 (28.5–50.0)	0.37
Sex, female *n* (%)	50 (70.4)	55 (77.5)	0.44
BMI (kg/m^2^), median (IQR)	23.0 (21.4–24.9)	23.6 (20.6–25.8)	0.99
eGFR (ml/min/1.73 m^2^), median (IQR)	98.2 (89.4–114.1)	98.9 (85.4–113.0)	0.52
Creatinine (mg/dl), mean ± SD	0.8 ± 0.1	0.8 ± 0.1	0.87
NfL (pg/ml), median (IQR)	5.3 (4.0–6.1)	5.0 (4.1–8.1)	0.48
GFAP (pg/ml), median (IQR)	55.1 (37.4–67.3)	61.4 (45.5–85.7)	0.016

Continuous variables are presented as median (interquartile range) or mean ± SD, and categorical variables as n (%). *P*-values were calculated using ANOVA for normally distributed continuous variables, Kruskal-Wallis test for non-parametric continuous variables, χ^2^ test for categorical variables, and Fisher’s exact test for proportional comparisons.

**Figure 4:**
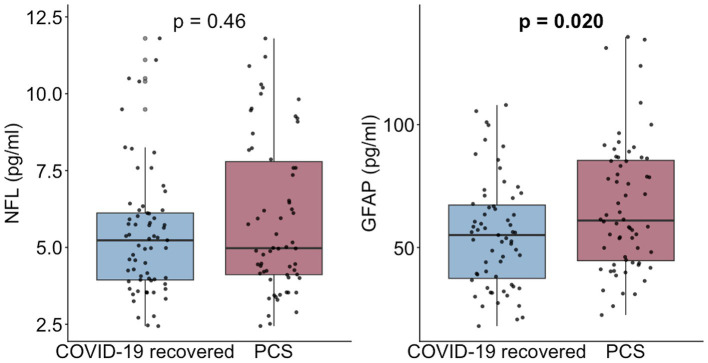
Serum NfL and GFAP concentrations in patients with PCS and recovered controls after age- and sex-matching. Boxplots display medians and interquartile ranges with individual data points overlaid. p-values derived from Wilcoxon test.

Distributions of NfL and GFAP after matching for age and sex are displayed in Figure 4. In the matched cohort, serum NfL did not differ between PCS and recovered controls (*p* = 0.48). In contrast, GFAP concentrations were higher in PCS than in controls (*p* = 0.016).

In the matched cohort, serum NfL levels did not differ between PCS patients and controls (*β* = 0.07, 95% CI −0.08 to 0.21, *p* = 0.348; Table 7). eGFR remained a significant predictor (*p* = 0.042). For GFAP, PCS patients showed slightly higher levels compared with controls (*β* = 0.17, 95% CI 0.02–0.32, *p* = 0.025). Again, eGFR was a significant predictor (*p* = 0.005).

**Table 7:** ANCOVA-style linear regression model after age- and sex-matching for log-transformed NfL and GFAP, adjusted for group (PCS vs. HC) and eGFR.

Predictors	Estimates	std. error	CI	Statistic	*p*
log(NfL)					
(Intercept)	2.06	0.22	1.63–2.50	9.35	**<0.001**
Group: PCS (vs HC)	0.07	0.07	−0.08 – 0.21	0.94	0.348
eGFR (ml/min/1.73 m^2^)	−0.00	0.00	−0.01 – −0.00	−2.06	**0.042**
Observations	115				
R^2^/R^2^ adjusted	0.046/0.028				
log(GFAP)					
(Intercept)	4.62	0.24	4.15–5.10	19.20	**<0.001**
Group: PCS (vs HC)	0.17	0.08	0.02–0.32	2.27	**0.025**
eGFR (ml/min/1.73 m^2^)	−0.01	0.00	−0.02 – −0.01	−2.90	**0.005**
Observations	116				
R^2^/R^2^ adjusted	0.115/0.099				

Coefficients are shown on the log scale (β) with 95% confidence intervals.

## 5 Discussion

### 5.1 Principal findings and interpretation

In this cross-sectional case–control study, we evaluated serum NfL and GFAP in patients with PCS compared with COVID-19-recovered controls after rigorous adjustment for age, sex, and renal function. Both biomarkers showed dependence on these covariates, underscoring their relevance as major confounders in PCS biomarker research. After multivariable adjustment, no statistically significant difference in NfL concentrations was observed between groups, indicating that apparent crude differences are largely explained by demographic and physiological factors rather than PCS status itself. In contrast, GFAP concentrations were modestly higher in PCS patients in an age- and sex-matched sensitivity cohort, suggesting a small and context-dependent signal that warrants cautious interpretation.

### 5.2 Comparison with existing literature

Our findings are consistent with recent clinic-based PCS studies reporting largely normal or age-adjusted NfL and GFAP concentrations. Boesl et al. (2024) observed biomarker values within reference ranges in PCS patients with cognitive complaints and found no association with neuropsychological performance, while Kanberg et al. (2024) reported normal cerebrospinal fluid and plasma biomarker levels during convalescence. Conversely, studies focusing on earlier post-acute phases or on more severe acute disease have described elevated NfL and GFAP (Peluso et al., 2022; Bark et al., 2023; Plantone et al., 2024), suggesting that timing of sampling and acute disease severity substantially influence biomarker levels.

Differences across studies likely also reflect variation in case definitions and adjustment for key confounders. Both NfL and GFAP increase with age (Polymeris et al., 2022; De Lorenzo et al., 2024) and correlate with renal function (Akamine et al., 2020; Polymeris et al., 2022; Vågberg et al., 2015; Koerbel et al., 2025; Axelsson et al., 2025). In line with this, we found that eGFR remained an independent predictor of GFAP in age- and sex-matched analyses, and that the modest GFAP difference between PCS and controls persisted after adjustment for renal function. Prior work in patients with chronic kidney disease demonstrated pronounced biomarker elevations once renal function is substantially impaired (Axelsson et al., 2025), whereas our cohort largely comprised individuals with preserved kidney function, indicating that mild eGFR variation within the normal range primarily acts as a confounder rather than a distinct pathophysiological driver.

### 5.3 Biological interpretation in PCS

The absence of a significant group difference in NfL argues against ongoing large-fiber axonal degeneration as a common mechanism underlying PCS. This finding is consistent with prevailing pathophysiological frameworks emphasizing microglial activation, neuroimmune dysregulation, endothelial dysfunction, and autonomic imbalance rather than active cytodestructive injury (Peluso and Deeks, 2024; Boesl et al., 2024; Kanberg et al., 2024; Greenhalgh et al., 2024). In this context, elevations of NfL would primarily be expected in settings of overt neuroaxonal damage or substantial blood–brain barrier disruption, as described during severe acute COVID-19 or early convalescence, but not necessarily in the chronic PCS phase. Consistently, exploratory analyses did not reveal significant correlations between PCS symptom severity scores and serum NfL or GFAP concentrations (Supplementary Figure 7), and additional adjustment for time since SARS-CoV-2 infection did not materially alter the multivariable results (Supplementary Tables 6, 7).

The modest increase in GFAP observed in the age- and sex-matched cohort may indicate low-grade or transient astroglial activation in a subset of patients. Given the small effect size and the sensitivity of GFAP to age and renal function, this signal should be interpreted cautiously and does not support a persistent astrocytic injury process. Astrocyte-mediated responses to systemic inflammation or vascular dysfunction may therefore remain below the detection threshold of GFAP, whereas biomarkers more closely linked to glial activation and neuroinflammation, such as YKL-40, a glycoprotein secreted by activated glial cells, may be better suited to capture subtle inflammatory processes proposed in PCS (Dichev et al., 2020).

Taken together, these findings support the concept of PCS as a heterogeneous syndrome in which neuroinflammatory or neurodegenerative mechanisms may characterize only a subset of patients, while non-destructive or systemic drivers of symptoms likely predominate and may not be adequately reflected by NfL or GFAP.

### 5.4 Strengths

This study has several notable strengths. It is among the first to systematically investigate serum NfL and GFAP in a well-characterized PCS cohort compared with rigorously defined COVID-19-recovered controls, using comprehensive adjustment for key physiological confounders. The cohort was established prospectively and characterized according to current consensus definitions with standardized clinical and symptom assessment, enhancing comparability and interpretability. By explicitly accounting for age, sex, and kidney function—known determinants of circulating biomarker levels—this study provides a methodologically robust framework for interpreting null findings. Inclusion of an age- and sex-matched sensitivity cohort further strengthened internal validity. The relatively long median interval since infection (>15 months) allowed assessment of biomarker status well beyond the acute phase, providing insight into long-term neurobiological recovery after SARS-CoV-2 infection.

### 5.5 Limitations

NfL is a sensitive marker of axonal injury but lacks specificity for the CNS and may reflect peripheral as well as central axonal damage (Bridel et al., 2019; Gafson et al., 2020). Circulating NfL concentrations are strongly influenced by age, renal function, systemic inflammation, and comorbid conditions, which may attenuate or obscure small disease-specific effects despite statistical adjustment (Fitzgerald et al., 2022; Koini et al., 2021). Similarly, elevations in GFAP do not necessarily indicate astrocytic injury but may represent reactive astrogliosis or transient glial activation in response to systemic or inflammatory stimuli rather than structural CNS damage (Yang and Wang, 2015). Beyond NfL, other biomarkers of axonal or neuronal injury such as total tau, or UCH-L1 have been proposed, but were not assessed in the present study (Domingues et al., 2024).

Importantly, serum NfL and GFAP primarily capture large-fiber axonal and astrocytic pathology and are relatively insensitive to subtle neuroimmune, synaptic, or microglial alterations proposed in PCS (Khalil et al., 2024). Consequently, normal or near-normal biomarker concentrations do not exclude biologically relevant, non-cytodestructive neurological alterations, which may require multimodal assessment approaches to be detected.

From a methodological perspective, the cross-sectional design precludes assessment of longitudinal biomarker trajectories or causal relationships. The single-center recruitment strategy and enrollment via a tertiary referral clinic and online announcements may have introduced selection bias toward individuals with more persistent or severe symptoms. In addition, the cohort was enriched for patients meeting criteria for ME/CFS, which may limit generalizability and contribute to heterogeneity within the PCS group.

Statistically, the moderate sample size limits power to detect small effect sizes and increases the risk of residual confounding. While age, sex, and renal function were explicitly accounted for, additional clinical variables were not included to avoid overadjustment or collider bias, and residual confounding by unmeasured factors cannot be excluded. The relatively low explanatory power of the regression models reflects the multifactorial nature of circulating NfL and GFAP rather than methodological inadequacy. Given the exploratory nature of the analyses, formal correction for multiple comparisons was not applied to the primary multivariable models, and results should be interpreted with emphasis on effect sizes and consistency across analyses rather than isolated *p*-values.

Future multicenter, longitudinal studies with broader phenotyping are warranted to validate these findings and to identify PCS subtypes with distinct neurobiological signatures.

### 5.6 Clinical and research implications

These findings indicate that serum NfL and GFAP have limited utility as standalone markers of persistent neurobiological injury in unselected PCS populations, particularly without rigorous adjustment for age and renal function. Importantly, the absence of robust group-level differences does not preclude potential relevance in specific clinical or biological subgroups, such as patients with prominent neurological manifestations or distinct inflammatory or vascular phenotypes. Future studies integrating neurological biomarkers with detailed clinical phenotyping, imaging, and immune or vascular markers may help identify subpopulations in which NfL and GFAP provide complementary clinical value.

## 6 Conclusion

In this cross-sectional case–control study, serum neurofilament light (NfL) concentrations did not differ between patients with PCS and recovered controls in either crude or adjusted analyses, arguing against ongoing neuroaxonal injury in this cohort. GFAP levels were modestly elevated in PCS in the age- and sex-matched cohort, and this difference persisted after adjustment for kidney function, indicating a small disease-related astroglial signal. Importantly, studies investigating NfL and GFAP in PCS or other conditions should rigorously account for confounding factors – especially age, sex and renal function – to avoid misattributing physiological variation to disease-related effects. PCS appears to represent a heterogeneous condition in which structural CNS injury is not a predominant or universal feature, although subtle or transient astroglial responses may occur in a subset of individuals. Future research should therefore emphasize longitudinal biomarker trajectories, multimodal integration with imaging and immune profiling, and the identification of clinical or molecular subtypes that may reveal distinct pathophysiological mechanisms.

## Data Availability

The raw data supporting the conclusions of this article will be made available by the authors, without undue reservation.
