# Supplementary material for: Serum NfL and GFAP in post-COVID syndrome: minimal evidence of CNS injury after adjusting for confounders
Source: Front Cell Neurosci. 2026 Mar 20;20:1750121. doi: 10.3389/fncel.2026.1750121 (PMC13046514; doi:10.3389/fncel.2026.1750121)
Supplement: Supplementary file 1 [file Data_Sheet_1.docx]

# Supplementary materials for

Serum NfL and GFAP in Post-COVID Syndrome: Minimal Evidence of CNS Injury After Adjusting for Confounders

Michael Wunderle^1^, Andrea Ribeiro^1^, Isabelle Lethen^1^, Rebecca Wicklein^2^, Emily Feneberg^2^, Anna Wöhnl^1^, Johanna Negele^1^, Veronika Kesseler^1^, Samuel Niedermayer^1^, Maciej Lech^3^, Timon Wallraven*^1^, Christoph Schmaderer*^1,4^

^1^TUM School of Medicine and Health, Department of Nephrology, TUM University Hospital, Technical University of Munich, 81675 Munich, Germany

^2^TUM School of Medicine and Health, Department of Neurology, TUM University Hospital, Technical University of Munich, 81675 Munich, Germany

^3^Medizinische Klinik Und Poliklinik IV, LMU University Hospital Munich, Ziemssenstraße 5, 80336, Munich, Germany

^4^German Centre for Infection Research (DZIF), Munich Partner Site, Munich, Germany

*These authors contributed equally to this work

# Data Preparation & Quality Control

## Data quality assessment of core variables

Histograms show raw and log-transformed distributions of serum NfL and GFAP, demonstrating substantial right skewness in the raw scale and improved symmetry after log-transformation (Supplementary Figure 1). Boxplots illustrate the presence of several higher-value observations, consistent with expected biological variability in these biomarkers. Missingness plots reveal moderate proportions of missing data (NfL 9.8%, GFAP 9.3%, eGFR 5.9%). Little’s MCAR test indicated that data were not missing completely at random (p = 0.016), suggesting a non-random mechanism. These findings justify the use of log-transformed NfL and GFAP values in subsequent analyses to better approximate normality. Outliers were retained in the primary models given their likely biological rather than technical origin, with sensitivity analyses planned to assess robustness. The observed non-random missingness underscores the importance of complete-case analyses accompanied by sensitivity checks using imputation approaches.

**Supplementary Figure 1:** Distributions of serum NfL and GFAP (raw and log-transformed), boxplots illustrating outliers, and missingness patterns of core variables. NfL (9.8%), GFAP (9.3%), and eGFR (5.9%) showed moderate missingness; Little’s MCAR test indicated that data were not missing completely at random (p = 0.016).

Influence plots for NfL and GFAP models identified a small number of observations with higher leverage (e.g., IDs 9, 71, 97, 110, 119, 142), but Cook’s distances remained below conventional thresholds, indicating no undue influence on overall model estimates (Supplementary Figures 2 and 3).

**Supplementary Figure 2:** Influence plot of the NfL regression model. Bubble size indicates Cook’s distance (influence), the x-axis represents leverage (hat-values), and the y-axis shows studentized residuals. Several observations displayed higher leverage (e.g., IDs 97, 110, 119), but Cook’s distances remained low, indicating no substantial influence on the model results.

**Supplementary Figure 3:** Influence plot of the GFAP regression model. Bubble size indicates Cook’s distance (influence), the x-axis represents leverage (hat-values), and the y-axis shows studentized residuals. A small number of observations exhibited higher leverage, but Cook’s distances remained below conventional thresholds, indicating no undue influence on model estimates.

Residual diagnostics supported the validity of the linear regression models for both NfL and GFAP. Residuals were symmetrically distributed without evidence of strong non-linearity or heteroskedasticity (Supplementary Figures 4 and 5). Q-Q plots indicated approximate normality of residuals, with only minor deviations in the tails. Scale–location plots suggested stable variance across fitted values. Cook’s distance plots identified a small number of potentially influential observations, but all values were below conventional thresholds, indicating no undue influence on model estimates. Robust standard errors (HC3) yielded results that were virtually identical to the primary models, confirming the stability of findings.

**Supplementary Figure 4:** Residual diagnostics for the NfL regression model. Panels display residuals vs fitted values, Q-Q plot of standardized residuals, scale–location plot, and Cook’s distance. No major deviations from model assumptions were observed.

**Supplementary Figure 5:** Residual diagnostics for the GFAP regression model. Panels display residuals vs fitted values, Q-Q plot of standardized residuals, scale–location plot, and Cook’s distance. Residuals were approximately normally distributed, and variance was stable across fitted values.

PCS participants exhibited a wide range of symptom severity. At the same time, both the PCS Score and the C19-YRS symptom severity score were centered in the moderate-to-severe range, indicating that the cohort predominantly comprised patients with substantial PCS symptom burden.

**Supplementary Figure 6:** Distribution of PCS symptom severity within the PCS cohort. Histograms show the distribution of the PCS Score (left) and the C19-YRS symptom severity score (right), illustrating a wide range of symptom severity among enrolled PCS participants.

**Supplementary Figure 7** shows exploratory Pearson correlation analyses between PCS symptom severity (PCS Score and C19-YRS symptom severity score) and serum NfL and GFAP concentrations within the PCS cohort. No significant correlations were observed.

**Supplementary Figure 7:** Pearson correlation analyses between PCS symptom severity and NfL and GFAP concentrations within the PCS cohort. Scatter plots show associations between PCS Score and NfL (top left), PCS Score and GFAP (top right), C19-YRS symptom severity score and NfL (bottom left), and C19-YRS symptom severity score and GFAP (bottom right).

# Sensitivity & Robustness Analyses

**Alternative renal metric**

Replacing eGFR with log(creatinine) produced virtually identical results (NfL: adjusted GMR 1.04 [0.91 – 1.18], p = 0.562; GFAP: 1.10 [0.97 – 1.26], p = 0.145), confirming that findings were not dependent on the choice of renal function estimator (Supplementary Table 1).

| Sensitivity: replace eGFR with log(creatinine) | | | | | |
| --- | --- | --- | --- | --- | --- |
| Analysis | **Marker** | **β (95% CI)** | **GMR (95% CI)** | **p-value** | **Adj. R²** |
| Alt renal metric (log creatinine) | NfL | 0.038 [-0.092; 0.168] | 1.04 [0.91; 1.18] | 0.562 | 0.048 |
| Alt renal metric (log creatinine) | GFAP | 0.098 [-0.034; 0.231] | 1.10 [0.97; 1.26] | 0.145 | 0.139 |

**Supplementary Table 1:** Sensitivity analyses replacing eGFR with log(creatinine). Group coefficients (PCS vs HC) are shown as β estimates on the log scale and exponentiated as geometric mean ratios (GMRs) with 95% confidence intervals.

**Non-linearity**

When modelling eGFR using restricted cubic splines (3 – 4 knots), the group coefficients remained non-significant for both biomarkers (NfL adjusted GMRs 1.03 – 1.04; GFAP 1.10 [0.96 – 1.26]), with no evidence of important non-linear effects (Supplementary Table 2).

| Sensitivity: eGFR modelled with restricted cubic splines | | | | | |
| --- | --- | --- | --- | --- | --- |
| Analysis | Marker | β (95% CI) | GMR (95% CI) | p-value | Adj. R² |
| Spline eGFR (3 knots) | NfL | 0.035 [-0.095; 0.165] | 1.04 [0.91; 1.18] | 0.600 | 0.043 |
| Spline eGFR (3 knots) | GFAP | 0.098 [-0.035; 0.231] | 1.10 [0.97; 1.26] | 0.149 | 0.133 |
| Spline eGFR (4 knots) | NfL | 0.032 [-0.099; 0.162] | 1.03 [0.91; 1.18] | 0.632 | 0.040 |
| Spline eGFR (4 knots) | GFAP | 0.097 [-0.036; 0.231] | 1.10 [0.96; 1.26] | 0.152 | 0.128 |

**Supplementary Table 2:** Sensitivity analyses modelling eGFR using restricted cubic splines (3 and 4 knots). Group coefficients remained non-significant.

**CKD exclusion**

Excluding participants with eGFR <60 mL/min/1.73 m² did not materially change results (NfL: GMR 1.03 [0.91 – 1.18], p = 0.610; GFAP: 1.10 [0.96 – 1.26], p = 0.151), indicating that results were not driven by participants with impaired kidney function.

| Sensitivity: exclude participants with eGFR <60 | | | | | |
| --- | --- | --- | --- | --- | --- |
| Analysis | Marker | β (95% CI) | GMR (95% CI) | p-value | Adj. R² |
| Exclude eGFR < 60 | NfL | 0.034 [-0.096; 0.164] | 1.03 [0.91; 1.18] | 0.610 | 0.041 |
| Exclude eGFR < 60 | GFAP | 0.097 [-0.036; 0.229] | 1.10 [0.96; 1.26] | 0.151 | 0.138 |

**Supplementary Table 3:** Sensitivity analyses excluding participants with eGFR <60 mL/min/1.73 m². Findings were unchanged.

**Age stratification**

Exploratory analyses by median age showed that in younger participants (≤34 years) GFAP was modestly elevated in PCS (GMR 1.21 [1.01 – 1.45], p = 0.036), whereas in older participants no differences were observed (NfL GMR 0.98, GFAP 1.00; both p ≈ 1.0). Stratification by age tertiles suggested potential group differences in the middle tertile (31 – 44 years, NfL GMR 1.36 [1.07 – 1.72], GFAP 1.40 [1.11 – 1.78]), but not in the youngest or oldest groups. These findings suggest no consistent age-modified effect across strata.

| Sensitivity: age-stratified models | | | | | | |
| --- | --- | --- | --- | --- | --- | --- |
| Analysis | Stratum | Marker | β (95% CI) | GMR (95% CI) | p-value | Adj. R² |
| Age stratified | Age ≤ 34 | NfL | 0.081 [-0.070; 0.232] | 1.08 [0.93; 1.26] | 0.291 | 0.052 |
| Age stratified | Age ≤ 34 | GFAP | 0.190 [0.012; 0.368] | 1.21 [1.01; 1.45] | 0.036 | 0.075 |
| Age stratified | Age > 34 | NfL | -0.022 [-0.246; 0.202] | 0.98 [0.78; 1.22] | 0.847 | -0.039 |
| Age stratified | Age > 34 | GFAP | -0.000 [-0.208; 0.208] | 1.00 [0.81; 1.23] | 0.999 | 0.054 |
| Age stratified | Age tertile 1 (≤30) | NfL | 0.000 [-0.169; 0.169] | 1.00 [0.84; 1.18] | 0.998 | 0.099 |
| Age stratified | Age tertile 1 (≤30) | GFAP | 0.123 [-0.085; 0.332] | 1.13 [0.92; 1.39] | 0.242 | 0.094 |
| Age stratified | Age tertile 2 (31–44) | NfL | 0.306 [0.067; 0.545] | 1.36 [1.07; 1.72] | 0.013 | 0.140 |
| Age stratified | Age tertile 2 (31–44) | GFAP | 0.339 [0.105; 0.574] | 1.40 [1.11; 1.78] | 0.006 | 0.254 |
| Age stratified | Age tertile 3 (>44) | NfL | -0.217 [-0.498; 0.064] | 0.81 [0.61; 1.07] | 0.128 | -0.004 |
| Age stratified | Age tertile 3 (>44) | GFAP | -0.201 [-0.457; 0.055] | 0.82 [0.63; 1.06] | 0.121 | 0.089 |

**Supplementary Table 4:** Age-stratified analyses (median split and tertiles). No consistent age-dependent group effect was identified.

**Multiple comparisons**

Univariate Mann–Whitney tests for group differences yielded p = 0.140 for NfL and p = 0.003 for GFAP (Supplementary Table 5). After Holm correction, NfL remained non-significant (p = 0.140) and GFAP remained significant (p = 0.005), consistent with the descriptive analyses.

| Univariate p-values with Holm adjustment | | |
| --- | --- | --- |
| Marker | **Mann-Whitney p** | **Holm-adjusted p** |
| NfL | 0.140 | 0.140 |
| GFAP | 0.003 | 0.005 |

**Supplementary Table 5:** Univariate Mann-Whitney tests with Holm-adjusted p-values for multiple comparisons.

To assess whether time since SARS-CoV-2 infection influenced serum NfL or GFAP levels, multivariable regression models were extended to include time since infection as an additional covariate. Inclusion of this variable did not materially change the association between group status and log-transformed NfL or GFAP concentrations (Supplementary Table 6 and 7).

| log(NfL) | | | | | |
| --- | --- | --- | --- | --- | --- |
| Predictors | **Estimates** | **std. Error** | **CI** | **Statistic** | **p** |
| (Intercept) | 1.71 | 0.34 | -Inf – Inf | 5.00 | **<0.001** |
| Group: PCS (vs RC) | 0.03 | 0.07 | -Inf – Inf | 0.39 | 0.694 |
| Age (years) | 0.00 | 0.00 | -Inf – Inf | 1.33 | 0.187 |
| Male (vs female) | -0.03 | 0.07 | -Inf – Inf | -0.41 | 0.685 |
| eGFR (ml/min/1.73 m²) | -0.00 | 0.00 | -Inf – Inf | -0.95 | 0.345 |
| time_since_infection (days) | -0.00 | 0.00 | -Inf – Inf | -0.65 | 0.514 |
| Observations | 167 | | | | |
| R^2^ / R^2^ adjusted | 0.058 / 0.029 | | | | |

**Supplementary Table 6:** Multivariable linear regression model for log-transformed serum NfL including time since SARS-CoV-2 infection.

| log(GFAP) | | | | | |
| --- | --- | --- | --- | --- | --- |
| Predictors | **Estimates** | **std. Error** | **CI** | **Statistic** | **p** |
| (Intercept) | 4.08 | 0.35 | -Inf – Inf | 11.54 | **<0.001** |
| Group: PCS (vs RC) | 0.08 | 0.07 | -Inf – Inf | 1.23 | 0.222 |
| Age (years) | 0.01 | 0.00 | -Inf – Inf | 2.10 | **0.037** |
| Male (vs female) | -0.14 | 0.07 | -Inf – Inf | -2.04 | **0.043** |
| eGFR (ml/min/1.73 m²) | -0.00 | 0.00 | -Inf – Inf | -1.39 | 0.168 |
| time_since_infection (days) | -0.00 | 0.00 | -Inf – Inf | -0.16 | 0.873 |
| Observations | 168 | | | | |
| R^2^ / R^2^ adjusted | 0.162 / 0.136 | | | | |

**Supplementary Table 7:** Multivariable linear regression model for log-transformed serum GFAP including time since SARS-CoV-2 infection.

In adjusted linear models of the full cohort, ME/CFS status was not associated with either biomarker (Supplementary Table 8). For NfL, the group coefficient was β = 0.10 (95% CI -0.03 to 0.24, p = 0.135; n = 158; adjusted R² = 0.048). For GFAP, β = 0.07 (95% CI -0.07 to 0.21, p = 0.331; n = 160; adjusted R² = 0.109). **Age** remained positively associated with GFAP (β ≈ 0.01 per year, p = 0.019) and showed a trend for NfL (p = 0.093). Gender showed a borderline association with GFAP (lower in males, p = 0.055), **eGFR** was not associated with either outcome.

|  | log(NfL) | | | | |
| --- | --- | --- | --- | --- | --- |
| Predictors | **Estimates** | **std. Error** | **CI** | **Statistic** | **p** |
| (Intercept) | 1.57 | 0.34 | 0.89 – 2.25 | 4.56 | **<0.001** |
| Group: ME/CFS | 0.10 | 0.07 | -0.03 – 0.24 | 1.50 | 0.135 |
| Age (years) | 0.01 | 0.00 | -0.00 – 0.01 | 1.69 | 0.093 |
| Male (vs female) | -0.03 | 0.07 | -0.17 – 0.12 | -0.35 | 0.726 |
| eGFR (ml/min/1.73 m²) | -0.00 | 0.00 | -0.01 – 0.00 | -0.73 | 0.469 |
| Observations | 158 | | | | |
| R^2^/R^2^ adjusted | 0.072 / 0.048 | | | | |
|  | **log(GFAP)** | | | | |
| Predictors | **Estimates** | **std. Error** | **CI** | **Statistic** | **p** |
| (Intercept) | 4.02 | 0.35 | -3.32 – 4.71 | 11.40 | **<0.001** |
| Group: ME/CFS | 0.07 | 0.07 | -0.07 – 0.21 | 0.97 | 0.331 |
| Age (years) | 0.01 | 0.00 | -0.00 – 0.01 | 2.37 | **0.019** |
| Male (vs female) | -0.14 | 0.07 | -0.28 – 0.00 | -1.93 | 0.055 |
| eGFR (ml/min/1.73 m²) | -0.00 | 0.00 | -0.01 – 0.00 | -1.07 | 0.287 |
| Observations | 160 | | | | |
| R^2^/R^2^ adjusted | 0.131 / 0.109 | | | | |

**Supplementary Table 8:** ANCOVA-style linear regression model for log-transformed NfL and GFAP, adjusted for group (ME/CFS vs no ME/CFS), age, gender, and eGFR. Coefficients are shown on the log scale (β) with 95% confidence intervals.
