# Supplementary figures and images for: Serum NfL and GFAP in post-COVID syndrome: minimal evidence of CNS injury after adjusting for confounders
Source: Front Cell Neurosci. 2026 Mar 20;20:1750121. doi: 10.3389/fncel.2026.1750121 (PMC13046514; doi:10.3389/fncel.2026.1750121)

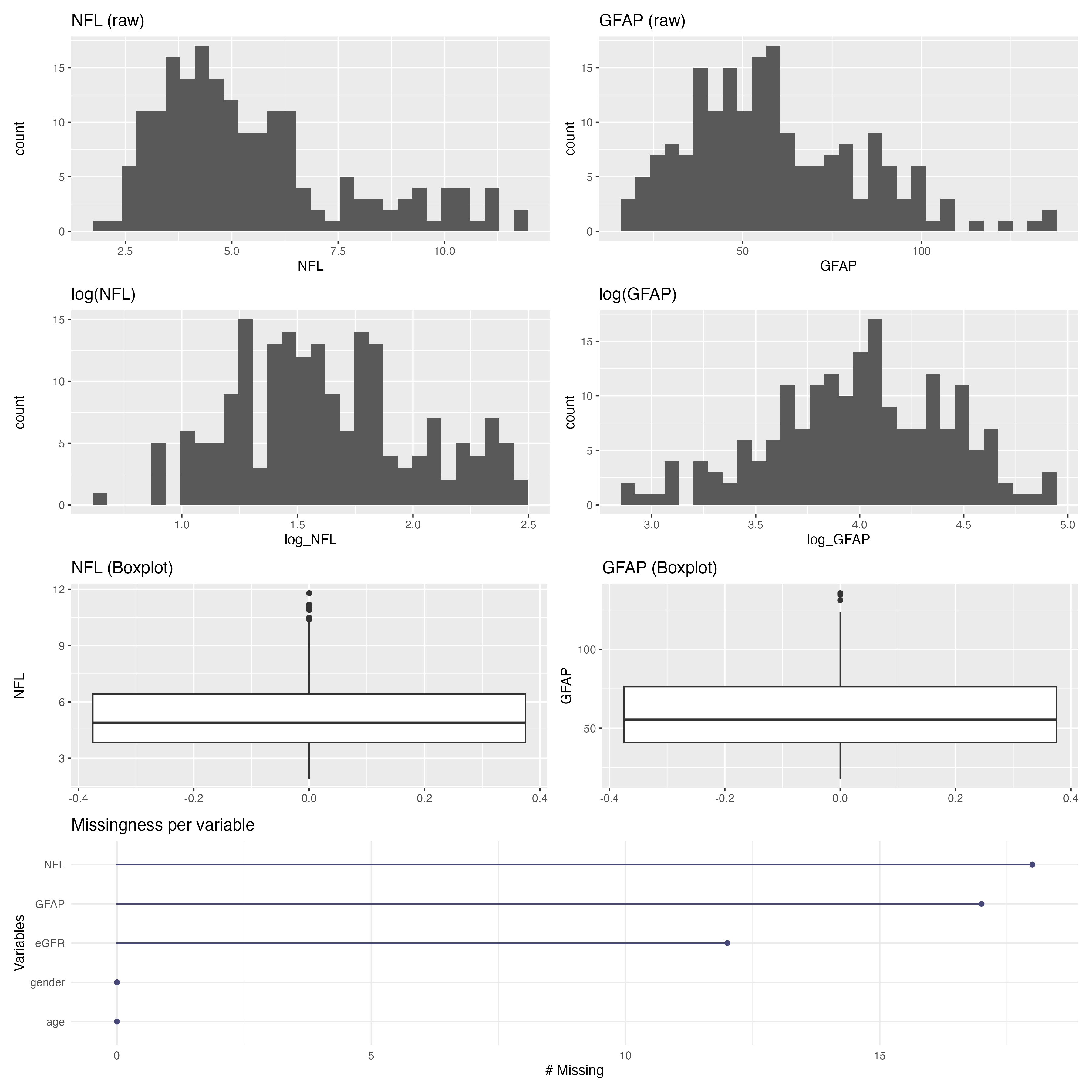

Supplement: Supplementary file 2 [file Image_1.png]

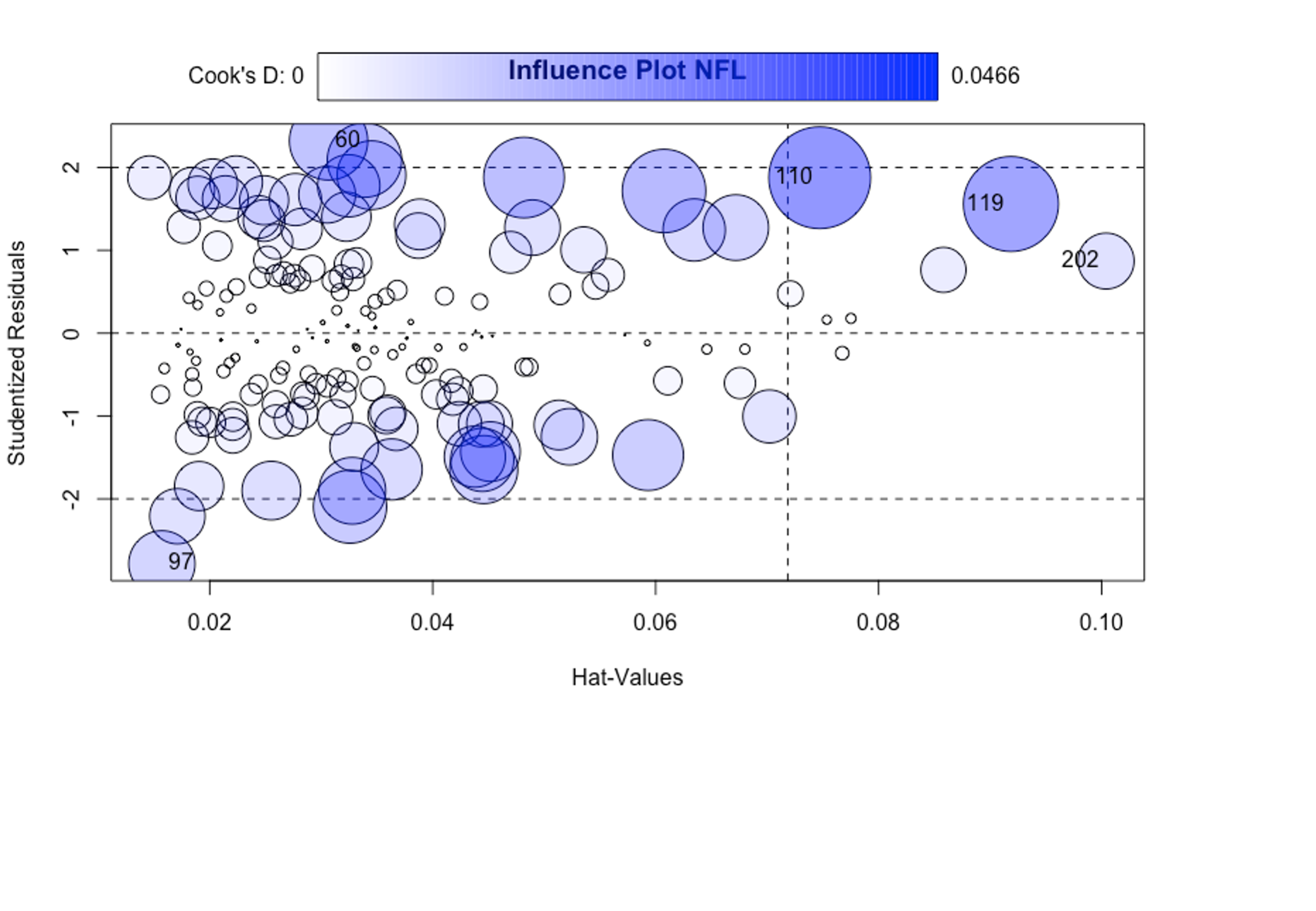

Supplement: Supplementary file 3 [file Image_2.png]

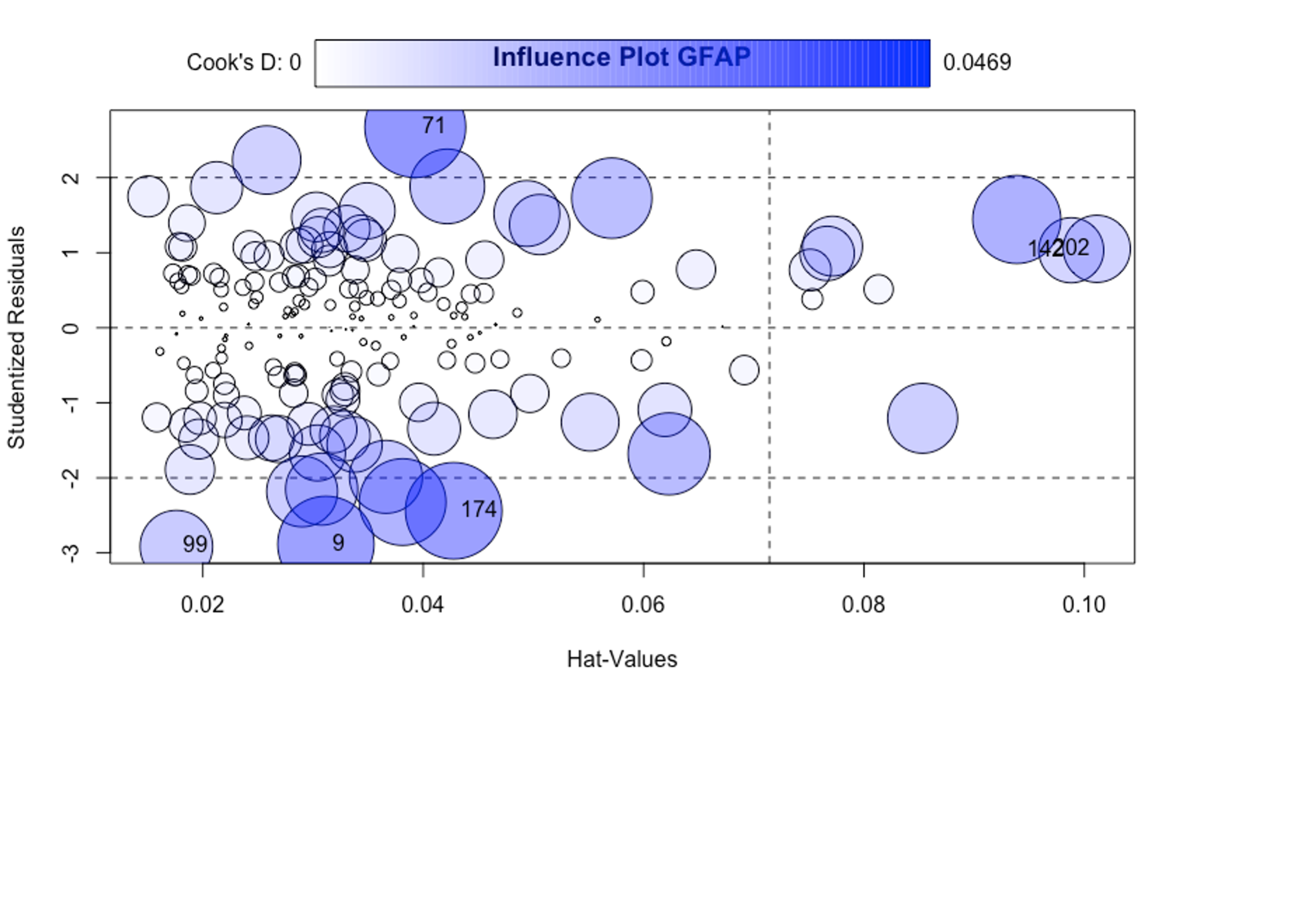

Supplement: Supplementary file 4 [file Image_3.png]

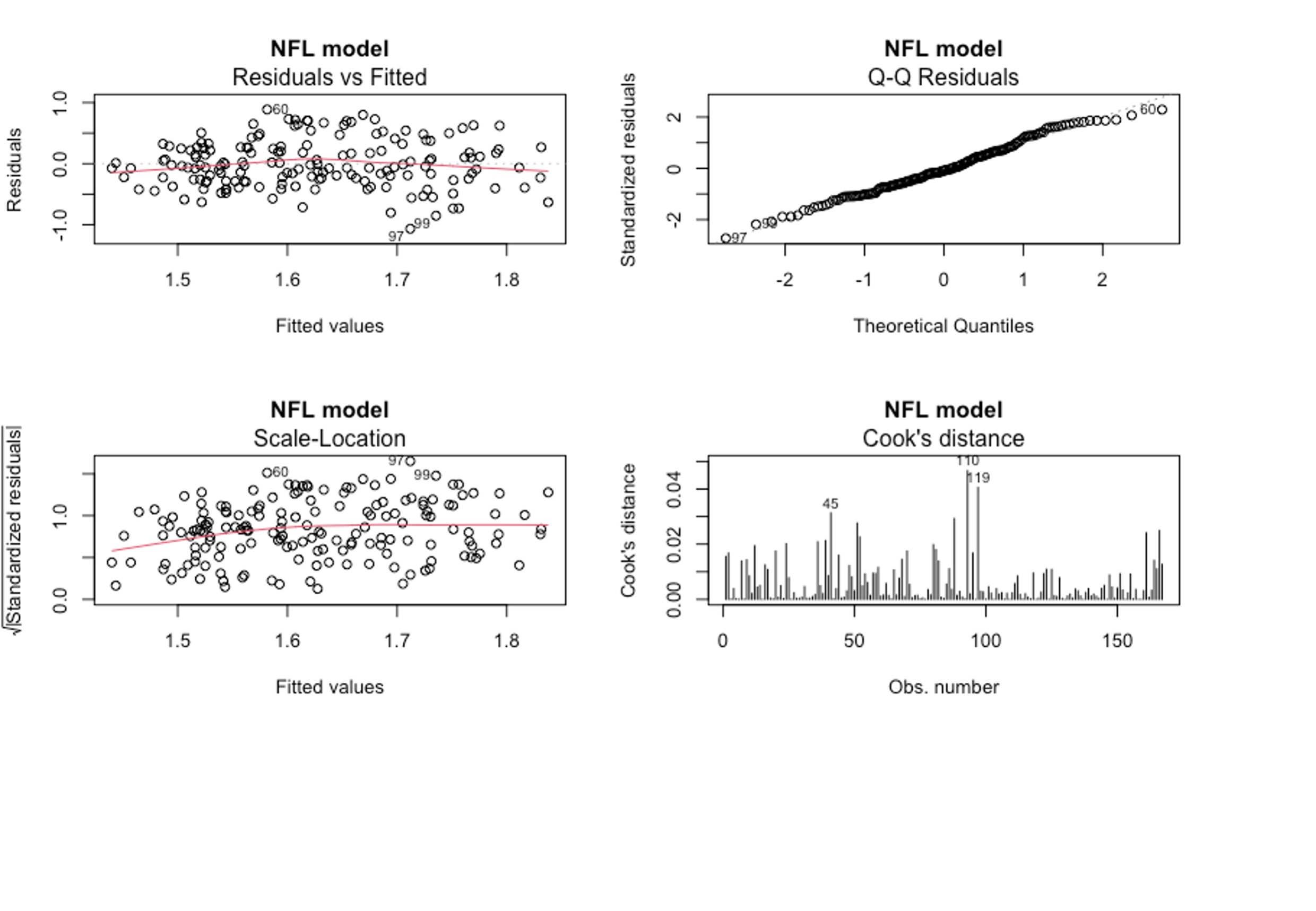

Supplement: Supplementary file 5 [file Image_4.png]

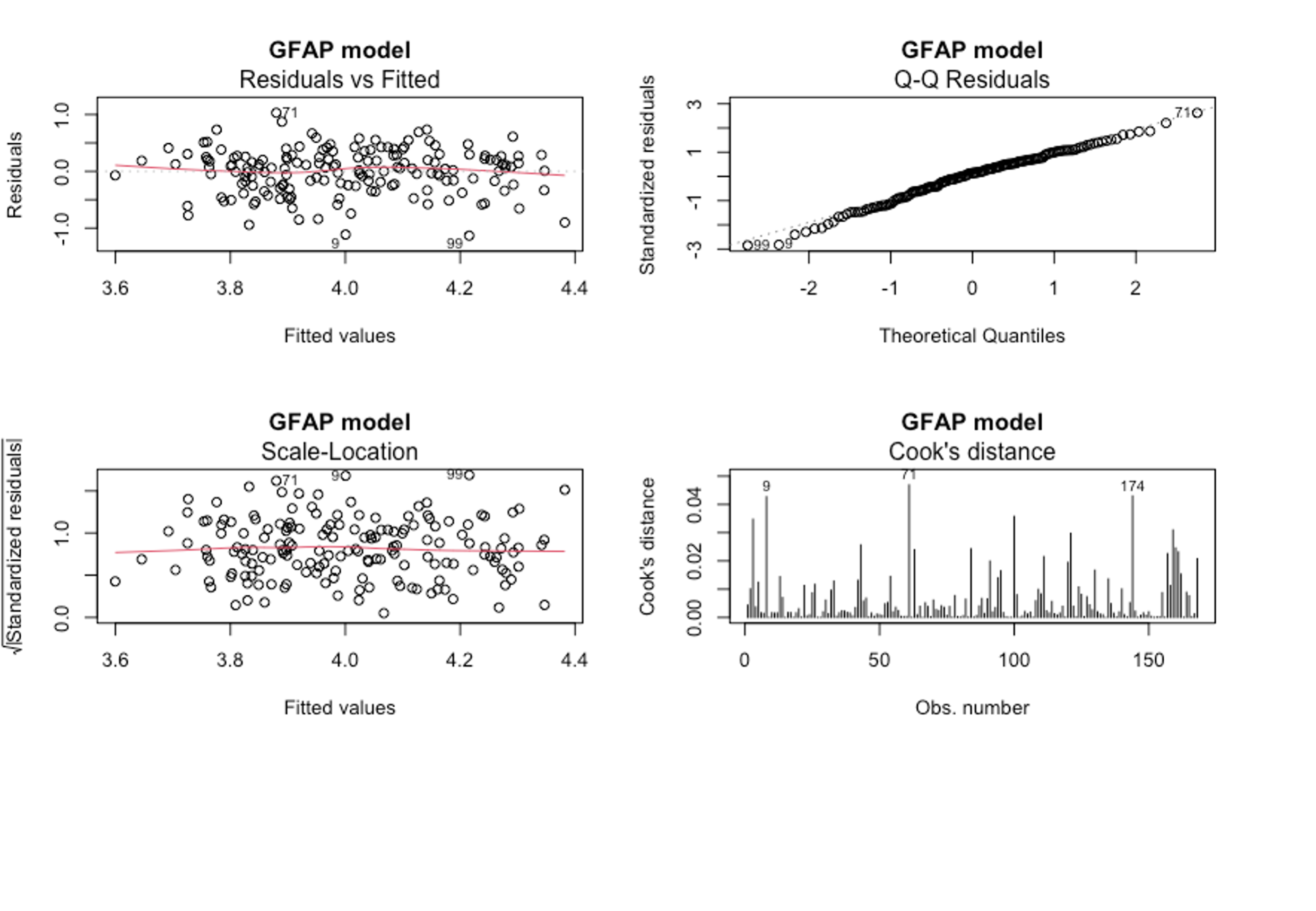

Supplement: Supplementary file 6 [file Image_5.png]

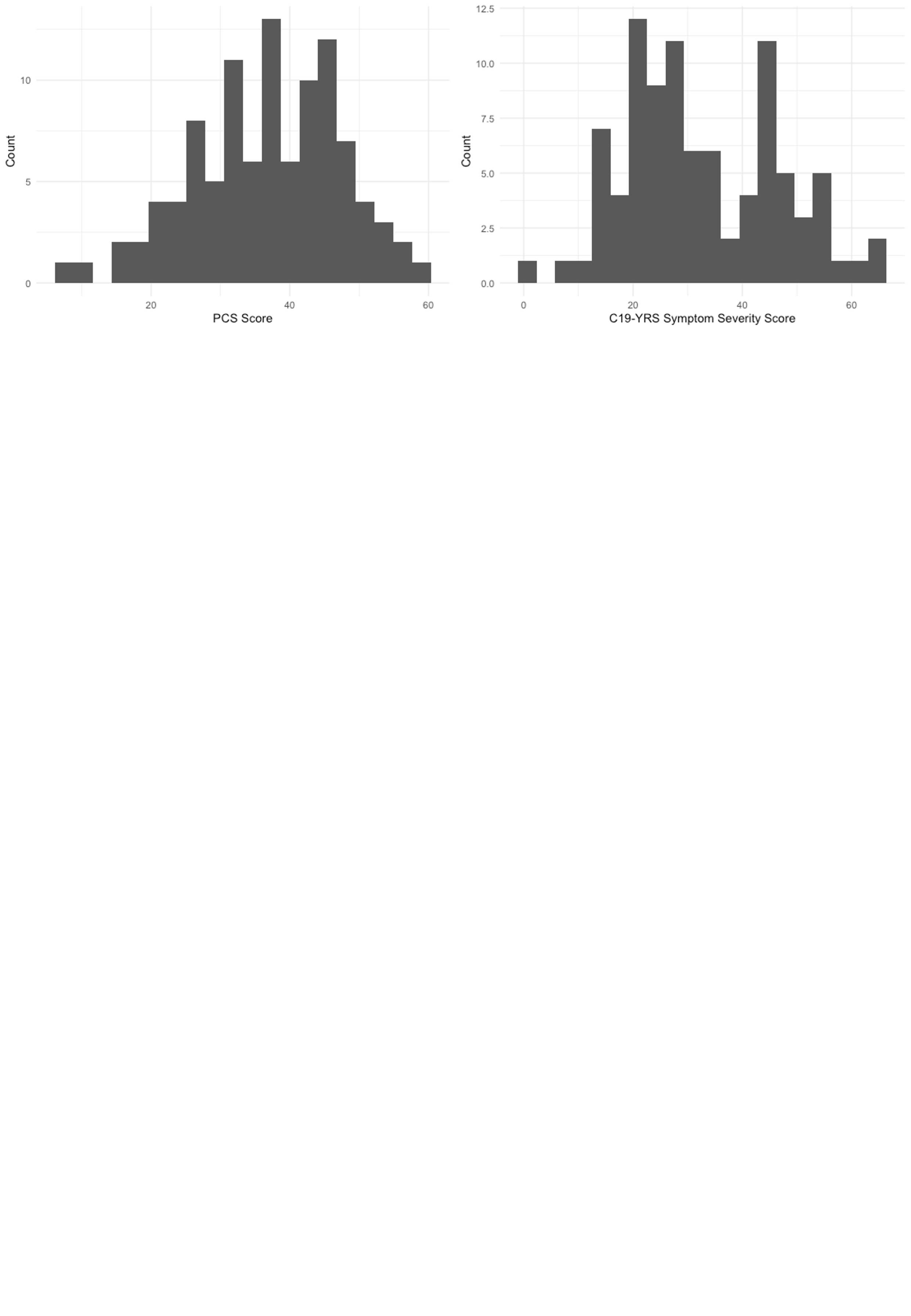

Supplement: Supplementary file 7 [file Image_6.png]

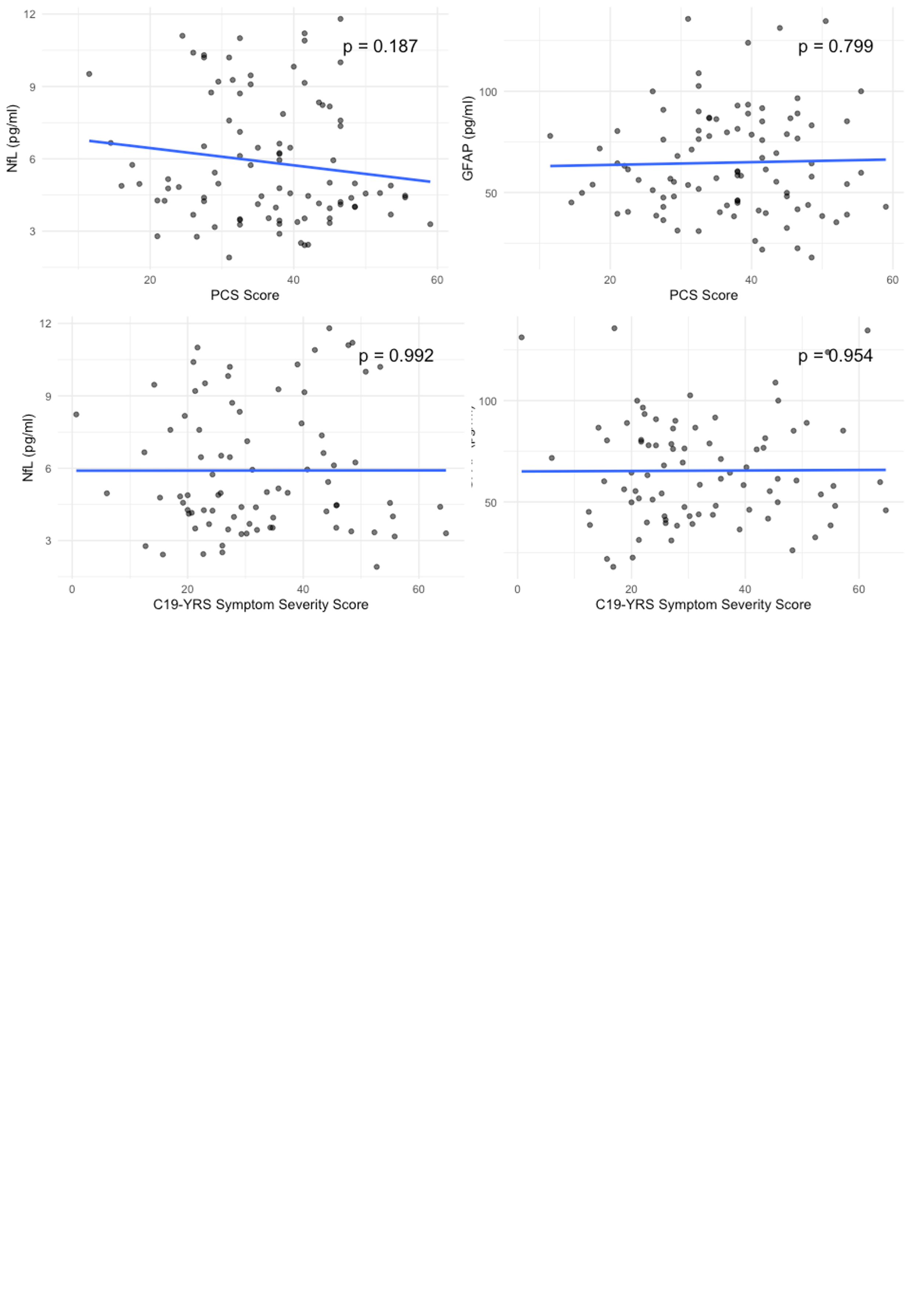

Supplement: Supplementary file 8 [file Image_7.png]
